# Machine Learning Model for Readmission Prediction of Patients With Heart Failure Based on Electronic Health Records: Protocol for a Quasi-Experimental Study for Impact Assessment

**DOI:** 10.2196/52744

**Published:** 2024-03-11

**Authors:** Monika Nair, Lina E Lundgren, Amira Soliman, Petra Dryselius, Ebba Fogelberg, Marcus Petersson, Omar Hamed, Miltiadis Triantafyllou, Jens Nygren

**Affiliations:** 1 School of Health and Welfare Halmstad University Halmstad Sweden; 2 School of Business, Innovation and Sustainability Halmstad University Halmstad Sweden; 3 School of Information Technology Halmstad University Halmstad Sweden; 4 Cambio Healthcare Systems AB Linköping Sweden; 5 Clinical Cardiology at Halmstad Hospital Halmstad Sweden

**Keywords:** artificial intelligence, machine learning, readmission prediction, heart failure, clinical decision support, machine learning model, CHF, congestive heart failure, readmission, prediction, electronic health records, electronic health record, EHR, quasi-experimental study, decision-making process, risk assessment, risk assessment tool, predictive models, predictive model, Sweden, physician, nurse, nurses, clinician, clinicians

## Abstract

**Background:**

Care for patients with heart failure (HF) causes a substantial load on health care systems where a prominent challenge is the elevated rate of readmissions within 30 days following initial discharge. Clinical professionals face high levels of uncertainty and subjectivity in the decision-making process on the optimal timing of discharge. Unwanted hospital stays generate costs and cause stress to patients and potentially have an impact on care outcomes. Recent studies have aimed to mitigate the uncertainty by developing and testing risk assessment tools and predictive models to identify patients at risk of readmission, often using novel methods such as machine learning (ML).

**Objective:**

This study aims to investigate how a developed clinical decision support (CDS) tool alters the decision-making processes of health care professionals in the specific context of discharging patients with HF, and if so, in which ways. Additionally, the aim is to capture the experiences of health care practitioners as they engage with the system’s outputs to analyze usability aspects and obtain insights related to future implementation.

**Methods:**

A quasi-experimental design with randomized crossover assessment will be conducted with health care professionals on HF patients’ scenarios in a region located in the South of Sweden. In total, 12 physicians and nurses will be randomized into control and test groups. The groups shall be provided with 20 scenarios of purposefully sampled patients. The clinicians will be asked to take decisions on the next action regarding a patient. The test group will be provided with the 10 scenarios containing patient data from electronic health records and an outcome from an ML-based CDS model on the risk level for readmission of the same patients. The control group will have 10 other scenarios without the CDS model output and containing only the patients’ data from electronic medical records. The groups will switch roles for the next 10 scenarios. This study will collect data through interviews and observations. The key outcome measures are decision consistency, decision quality, work efficiency, perceived benefits of using the CDS model, reliability, validity, and confidence in the CDS model outcome, integrability in the routine workflow, ease of use, and intention to use. This study will be carried out in collaboration with Cambio Healthcare Systems.

**Results:**

The project is part of the Center for Applied Intelligent Systems Research Health research profile, funded by the Knowledge Foundation (2021-2028). Ethical approval for this study was granted by the Swedish ethical review authority (2022-07287-02). The recruitment process of the clinicians and the patient scenario selection will start in September 2023 and last till March 2024.

**Conclusions:**

This study protocol will contribute to the development of future formative evaluation studies to test ML models with clinical professionals.

**International Registered Report Identifier (IRRID):**

PRR1-10.2196/52744

## Introduction

Care for patients with heart failure (HF) causes a substantial load on the health care system. One of the prominent challenges associated with HF care is the elevated risk of readmissions within 30 days following initial discharge [1]. While this readmission risk underscores that patients receive life-saving care, it also encompasses implications of health care costs, patient’s stress, and the impact of socioeconomic determinants on care outcomes [2]. The risk of readmission due to the worsening of HF symptoms is heightened by inappropriate treatment strategies, infectious complications, or prematurely executed discharges. Therefore, readmissions can be reduced by taking steps both during admission and hospitalization and post discharge to ensure compliance with care plans and improved treatment outcomes.

In current practice, clinicians make expert decisions, weighing in the probability of readmission of a patient by evaluating clinical data such as a patient’s medical history, medication list, laboratory tests as well as social factors [3]. However, the process of assessing a patient’s readiness for discharge introduces an element of subjectivity and uncertainty. Questions surrounding the probability of readmission emerge, prompting deliberation on optimal timing for discharge—whether immediately or with a slight delay. Additionally, the patient’s social context plays a critical role; decisions must be made regarding the suitability of home care versus outpatient clinic assignment.

In pursuit of curbing health care costs and mitigating uncertainty novel risk assessment tools, often in the form of predictive models have been developed. Drawing upon statistical, conventional machine learning (ML), and deep learning methodologies, these tools are designed to identify patients at risk of hospital readmission [4-6]. Leveraging risk indicators such as age, illness severity, prior hospitalizations, and other factors, these models predict the likelihood of readmission within a specific time frame. Preventive approaches can then be developed and applied to target the identified high-risk patients. The profound potential for cost savings within the health care domain has fueled substantial interest in rigorous testing and validation of similar models, underscoring the imperative of optimizing patient care while securing resource efficiency.

In this project, our objective is to evaluate the applicability and potential benefits of a previously established ML model for predicting unscheduled readmission of patients with HF within 30 days after discharge from medical care [5]. This model has been further fine-tuned and tailored for practical integration and usage within clinical settings [3,7], encompassing the identification of potential barriers and enablers for implementing a clinical decision support (CDS) tool presenting the model output for clinical use. Refinement also encompassed the augmentation of the model with interpretability features and a comprehensive exploration of the optimal timing and manner in which the model’s findings should be presented to users within the clinical domain [7]. The primary aim of the study outlined in this protocol is to investigate how the developed CDS tool alters the decision-making processes of health care professionals in the specific context of discharging patients with HF, and if so, in which ways. Additionally, the aim is to capture the experiences of health care practitioners as they engage with the system’s outputs to analyze usability aspects and obtain insights related to future implementation.

## Methods

### Study Design

The design of this study used the principles of Template for Intervention Description and Replication [8] to support clarity of the description of the intervention and the replicability of its implementation. This study has a quasi-experimental design with a randomized controlled crossover assessment of 2 groups of physicians and nurses working in pairs. This study will be conducted within the health care settings of 1 region in Southern Sweden. The 2 groups will be presented with patient scenarios using purposeful sampling [9] on which they are to make decisions on subsequent care plans and treatment strategies.

### HF Care Setting

An HF patient’s referral to the hospital can occur through the emergency department, primary care, or home care services. Upon arrival, the patient is allocated to one of the medical departments within either of 2 hospitals in the included region possessing specialized cardiology units. Once admitted, a comprehensive care plan is made, considering the patient’s symptoms, prior diagnoses, and relevant test results. This care plan also includes decisions regarding the appropriate timing for discharge. Within the framework of this study, this in-patient scenario, as detailed in a prior work [3], serves as the setting for HF care in which our investigation into the application of the CDS model takes place.

### Preparation for This Study

#### CDS Model

The model is based on comprehensive retrospective electronic health data in a Swedish region [10]. The cohort used for the development consisted of patients diagnosed with HF according to the *ICD-10* (*International Statistical Classification of Diseases, Tenth Revision;* I10.0, I42, I43, and I50), were residents, and receiving care in the region. The patients included were aged ≥40 years and had at least one admission after being diagnosed with HF between January 1, 2017, and December 31, 2019. All-cause hospitalization was considered. For each admission in the cohort, all patient’s previous admissions within 5 years were considered from the time of admission as the medical history of the patient (look back period); these admissions are not considered as events in this study but were used only as historical data.

Besides demographic information, variables were collected out of electronic health data related to different categories. These categories can be detailed as follows: comorbidities in which patient conditions related to HF are traced back, for example, hypertension, diabetes, chronic kidney disease, and atrial fibrillation. Diagnoses (including procedures) and medications in the electronic health data system were represented according to standard schemas: *ICD-10-SE* (*Swedish version of the 10th revision of the International Classification of Diseases*) and Anatomical Therapeutic Chemical codes, respectively. Laboratory results were also used, including specific features for some laboratory tests, such as N-terminal prohormone of brain natriuretic peptide, sodium, potassium, ferritin, and estimated glomerular filtration rate. Variables were defined to indicate the level of abnormality in the obtained results of these laboratory tests. Following clinical feedback on model development, features associated with a patient’s vital signs collected during the admission such as weight, heart rate, and blood pressure were also considered.

A conventional ML model was developed with *CatBoost*, which resembles gradient-boosting decision trees. The *CatBoost* model makes predictions using a series of decision trees, representing an explainable model [11]. In this study, the *CatBoost* python package (version 1.0.4) was used. The model was trained using a stratified 10-fold cross-validation, such that the training data were further divided into 10 parts where 90% (14,069) of data were used for training and 10% (1842) used for validation. Evaluation of the performance is based on commonly used performance measures such as sensitivity, specificity, *F*_1_-score, receiver operating characteristic curves, the area under the receiver operating characteristic curve, and the area under the precision-recall curve. The performance of the model is presented in a retrospective ML study [7].

The Shapley Additive Explanations (SHAP) technique was adopted to provide more details behind the model decision regarding important features for readmission prediction [12]. SHAP works as a model-agnostic explanation tool and provides local (ie, patient-specific) as well as global explanations (ie, across patient cohorts). SHAP was used to compute explainability outputs for the selected patient scenarios, which were later used as input to the CDS tool. For each prediction, the most important features were listed that were positively or negatively driving toward the readmission risk. The explanations provided were assessed by physicians for clinical relevance.

#### Stakeholder Study

To prepare for the experiment, an interview study was performed to determine the potential barriers and facilitating factors for the implementation of the CDS tool. In total, 12 interviews with stakeholders were performed in a Swedish health care organization consisting of 2 hospitals, primary care, and partial home care. The views on the CDS tool were collected from different roles such as medical process leaders, medical specialists in cardiology, specialist nurses, physiotherapists, home care physicians, home care nurses, and administrative roles [3]. Interviews were transcribed and thematically analyzed to condense and categorize content.

#### Explainable Artificial Intelligence Design Process

A design process was carried out to create the user interface of the CDS tool. The initial phases included expert interviews, care process observations, and literature searches about the design of explainable artificial intelligence (AI) systems. There are several design problems to address in creating an interface for such a decision support system, for example, to understand who the intended users are and their needs, what information flows the system shall interact with, and in what part of the care process such information is relevant [13]. Therefore, an iterative design process was adopted, to move forward step by step, while generating and testing different design ideas.

Initial usability tests were conducted after generating a set of low-fidelity prototypes. In total, 5 clinicians (3 physicians and 2 nurses) were individually testing the different prototypes over recorded video calls. The tests evaluated if the prediction score and the explanation presented in the prototypes were understandable, and how clinicians perceived this information given the situation of an AI-model as the source. The tests gave important information on which language to use and what to display in the interface, which was the focus of the next iteration. The initial usability tests further raised questions about where this tool might be needed and who the users could be. Consequently, additional care process observations were made, which led to some changes to the definition of the target users and the place of potential implementation.

All usability test data were analyzed, categorized, and used as input for the further development of the prototype. Thereafter, additional usability testing was performed, following the same procedure as previous tests, however, focusing on exploring how to present more detailed information. Another 5 clinicians (three of them participated in the first usability tests) tested these prototypes over video calls. The outcome of this second round of usability tests was used as input to another design update before a high-fidelity prototype was created. This prototype was tested with 4 clinicians (2 physicians and 2 nurses who also participated in the previous tests).

As the last step of the prototype development, a series of design workshops were conducted with user experience resources, to finalize the prototype and set the details, before a smaller usability test was performed with 2 clinicians (1 physician and 1 nurse). Their input led to some minor interface changes, and now the development of the CDS tool could begin.

#### Scenario Creation

From the same data set used to develop the CDS model, 20 patients were selected to constitute the target population in this study by using stratified random sampling. The stratification was made to ensure that there are equal amounts of readmitted patients and control patients. For each of these 20 patients, scenarios comprising narrative reports and clinical data describing demographics, and clinical events and outcomes, including potential readmittance, were recreated retrospectively based on real patient historical data from the regional database of electronic health data.

### Recruitment

The participants involved in the experimentation will include both physicians (n=6) and nurses (n=6) and will be recruited from the clinical staff at the 2 hospitals based on the following inclusion criteria: a minimum of 3 years of experience in the treatment of patients with HF, and currently active engagement in clinical practice within either of the 2 specialized cardiology departments in the region. This study will use purposeful sampling [9] for recruiting physicians and nurses according to the inclusion criteria, a process led by the hospital. Although there might be a limited availability of staff, physicians and nurses who were involved in the design of this study and the CDS model will be excluded from participation in this study, to prevent bias.

### Experimentation and Data Collection

**Figure 1:**
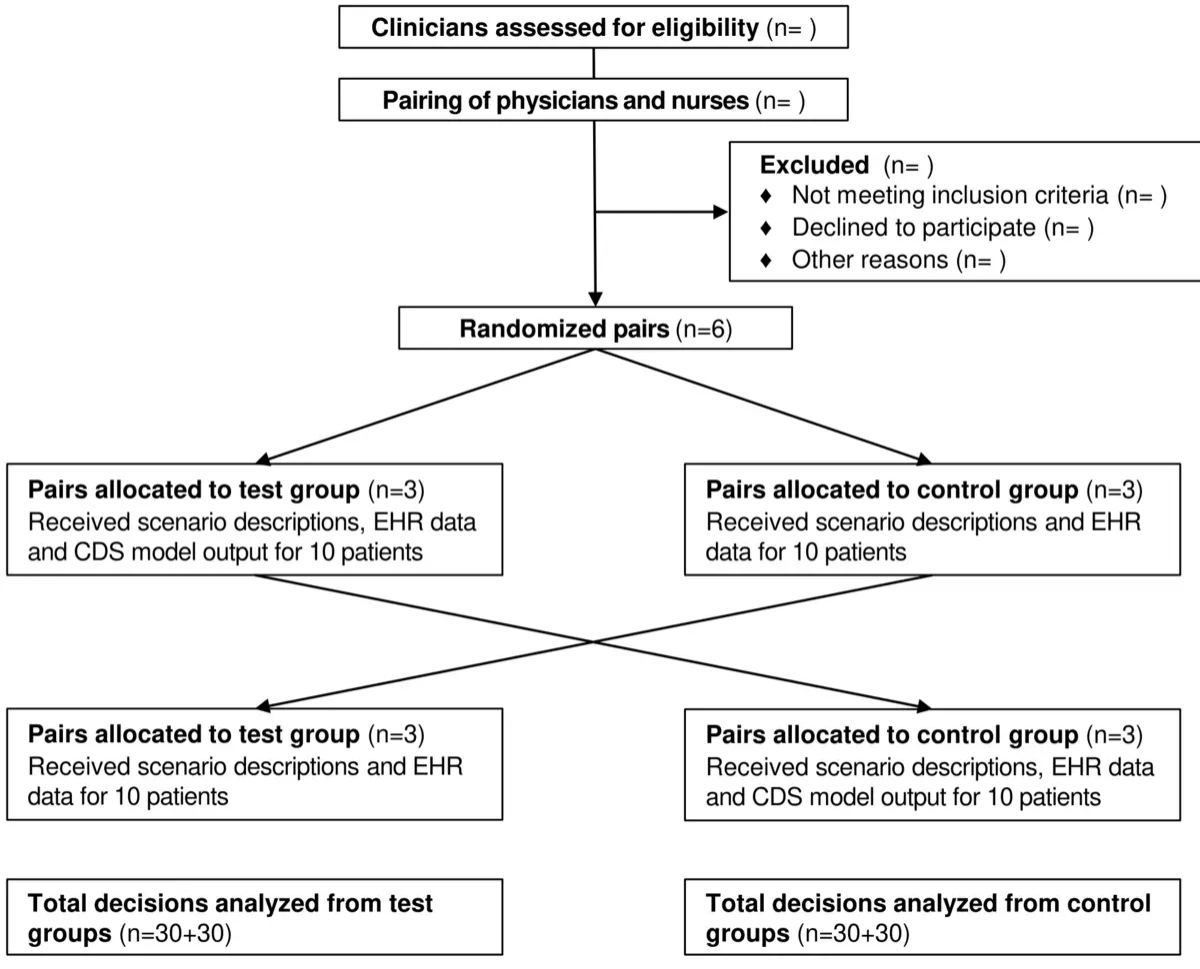
Flowchart for the randomized controlled cross-over design. CDS: clinical decision support; EHR: electronic health record.

The experiment simulates the decision-making process of a patient’s discharge, further treatments, and care plan, based on real historical data. Patient data for model output are preloaded into the CDS model. The participants will be randomly paired with 1 physician and 1 nurse in each pair, reflecting the real-world setup in the decision-making regarding the patient’s potential readmission (Figure 1). Pairs will then be randomly assigned into test (n=3) and control (n=3) groups using simple randomization with stratification. The pairs in the test group will be given 10 scenario descriptions, patient electronic health record (EHR) data, and the CDS model output based on each of the 10 patients. The pairs in the control group will be given the same 10 scenario descriptions and EHR data but not the CDS model output. After making decisions based on the 10 scenario descriptions the test group and the control group will switch roles and 10 new scenario descriptions will be given to the groups, again with and without the CDS model output.

**Figure 2:**
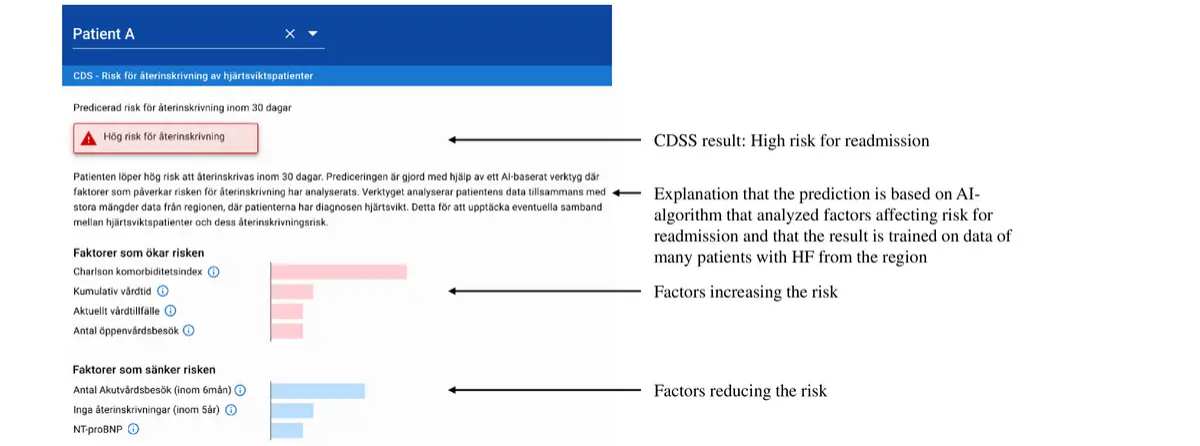
CDS model output showing readmission risk and explanations of model decision for a hypothetical patient. AI: artificial intelligence; CDS: clinical decision support; CDSS: clinical decision support system; HF: heart failure.

Before the experiment, each group will be briefly trained in how the CDS model works and how to interpret its output [14,15]. The clinicians will be asked to think aloud so that both the conversation and reasoning during the decision process are recorded. The scenarios will be presented to the clinicians via a text document in a separate computer room at the hospital, and the CDS output in a web-based interface as depicted in Figure 2. The clinicians will be able to either make a decision or request for more information about the patient, which will be provided by an additional clinician who is part of the research group that will be in another room using a chat function on the local information system. This clinician is connected to the EHR system and has access to all patient data. The additional information can include admission notes, laboratory test results, and medication lists, and this procedure will be carefully explained to the clinicians before the experiment. This information will not be available from the beginning, to mimic the real-world information search between different systems or system modules and to research what level of information would be sufficient to decide in both groups; a clinician shall provide extra information about the patient only if requested. Every such request will be documented by the researchers present in the rooms together with the clinicians, observing the decision-making and the experiment. All clinical notes available in the experiment will undergo a deidentification process before this study. The decisions made by the participants will be noted by the observing researcher.

At the end of the experiment, each clinician will be interviewed individually by the researcher using the interview questions defined in Multimedia Appendix 1. The dialogue will be audio-recorded and transcribed verbatim.

### Outcome Measures

A research-based evaluation framework specifically designed for AI-based decision-support systems [16] has guided and inspired the selection of the outcome measures for this study. The added value of the AI system will be assessed through a mixed methods evaluation design: process efficiency and patient-related outcomes shall be assessed using a quantitative approach, while quality, reliability, trust, and similar parameters shall be addressed through qualitative semistructured interviews. Table 1 provides details on the outcome measures selected for this study.

**Table 1:** Outcome measures.

Outcome indicator		Goal	Analytic approach
**Impact on decision-making**			
	Decision consistency	To explore decision consistency between the users and the CDS^a^ model to research whether having the CDS model increases uniformity among the decisions taken compared to decisions without the model.	Comparing the taken decisions: Between the CDS model output and the test group to assess the extent of how much the users agreed with the model output. Between test and control groups to assess similarity in decisions taken with and without the model output. Potential decisions: To discharge, with x, y, and z treatment. Move to another department (which?). Take additional tests for an extended investigation. To send a referral or follow-up at outpatient clinical, primary care, or home care. To stay for some more days.
	Decision quality	To explore whether the CDS model could improve decisions.	Comparing decisions taken by the test group with: Historical data—what decision was actually taken for the patient and what event followed afterwards. Control group’s decisions on the same patients to control the influence of the experiment and set up over the decisions taken: do decisions differ from the test group and the historical data?
	Work efficiency	To explore performance changes due to using the CDS model output.	Measuring speed of decision-making for both test and control groups. Comparing the average speed between groups with and without CDS.
**Impact on experience with decision-making**			
	Perceived benefits	Clinicians’ attitude toward perceived benefit for patients and clinicians of AI^b^.	Interview with nurses and physicians who were exposed to the CDS model output (Multimedia Appendix 1, Q1).
	Knowledge	Knowledge sufficiency and possible gaps.	Interview with nurses and physicians who were exposed to the CDS model output (Multimedia Appendix 1, Q5).
	Confidence	Confidence in making decisions using the algorithm (trust in the algorithm and data, and self-confidence).	Asking the participants in the test and control groups to take decisions for some patients with and without the CDS model output. Clinicians will be asked to rate their decision confidence and indicate to what extent the CDS output helped them using a 5-point Likert scale (1=not at all, 5=a great deal) [16]. Interview with nurses and physicians who were exposed to the CDS model output (Multimedia Appendix 1, Q3 and Q7).
	Reliability and validity	How reliable and valid are the suggestions by the algorithm—perception?	Interview with nurses and physicians who were exposed to the CDS model output (Multimedia Appendix 1, Q3).
	Perceived service quality	How is the perception of the overall clinician-provided service perceived?	Interview with nurses and physicians who were exposed to the CDS model output (Multimedia Appendix 1, Q2 and Q6).
	Unintended consequences	Unintended consequences are foreseen.	Interview with nurses and physicians who were exposed to the CDS model output (Multimedia Appendix 1, Q4).
	Intention of use	Obtaining an indication of worthiness to continue developing the AI-based system.	Interview with nurses and physicians who were exposed to the CDS model output (Multimedia Appendix 1, Q1, Q2, and Q6).
**Implementation aspects**			
	Workflow integration	How integrable is the solution into the current workflows?	Interview with nurses and physicians who were exposed to the CDS model output (Multimedia Appendix 1, Q4 and Q8).
**Usability**			
	Perceived ease of use	Perception of the features, human-computer interface.	Interview with nurses and physicians who were exposed to the CDS model output (Multimedia Appendix 1, Q5).

^a^CDS: clinical decision support.

^b^AI: artificial intelligence.

### Data Analysis

The quantitative analysis will primarily employ descriptive statistics. The interview data to address the rest of the outcome measures shall be transcribed and thematically analyzed using the thematic coding scheme corresponding but not limited to the outcome measures of this study (Table 1) [16-18].

### Ethical Considerations

This study conforms to the principles outlined in the Declaration of Helsinki and will fulfill the following requirements for research: information, consent, confidentiality, and safety of the participants, guided by the ethical principles of autonomy, beneficence, nonmaleficence, and justice. The anonymized patient data used in this study are available (2022-07287-02).

Patients whose data will be used for creating the scenarios will be informed through an advertisement on the university web pages and provided with the possibility to express a will to opt out of this study. The absence of an objection to the use of personal medical data in the research process will be considered as consent to participate.

All participants will receive written and oral information about the studies in which they are directly or indirectly involved. Participants will also be given information about the voluntary nature of the studies, confidentiality, and the ability to withdraw their consent at any time without having to justify why. All personal data will be registered according to the General Data Protection Regulation (GDPR2016/679) and the data will be stored per the Archive Act in Sweden (SFS1990:782).

## Results

The project is funded by the Knowledge Foundation and is part of the Center for Applied Intelligent Systems Research Health research profile (280042), which started in July 2021 and ends in 2028. The profile conducts research projects in coproduction with industry about the development, design, and implementation of AI systems in health care. The project HF readmission prediction started in July 2021 and will end in June 2024.

The recruitment process will start in September 2023 and last until March 2024. First, the testing and fine-tuning of this study process using the prototype shall be conducted (planned for September 2023). Then, data will be collected and analyzed during the experiment from October 2023. This study’s results are expected to be published. This research will involve several partners: Halmstad University (Sweden), Cambio AB (Sweden), and 1 Swedish region. This research aims to reduce avoidable readmissions of patients with HF.

## Discussion

### Principal Findings

This study focuses on the ML model for predicting unscheduled readmission of patients with HF within 30 days of discharge [5]. The purpose of the study described in this protocol is to assess the impact of this ML model on decision-making by health care professionals and to capture their experiences in using the model.

A quasi-experimental study shall be conducted with clinicians using the model and results shall be compared against a control group. Throughout the experiment, the researchers shall observe the decision-making process, take measurements, and collect feedback. Such knowledge shall increase an understanding of the potential impact on the decision-making, usability, perceived value if such a model is deployed, and willingness to use such tools in the future. Furthermore, this study will give practical insights into the factors that will potentially influence implementation that could be further used in the implementation process. In addition, concrete outcomes’ measures are suggested which can assist in future developments of similar models.

This study has several limitations. First, although the CDS model output shall be provided as a digital interface, the system is not in this version integrated with EHR and it will not be possible to check the actual impact of the CDS model on the workflow. Second, the samples of admissions will be selected using stratified sampling where subgroups will be created for readmission labels assigned to patient encounters. The readmission rate in our data set is 21% (3334 out of 15,911 admissions), thus the chance to obtain enough examples where the readmission label is true will be very low. Accordingly, stratification will be used to divide the population data into 2 subgroups before sampling, then select 10 cases having true as a value for readmission label. Third, this study shall not assess the impact of the use of the model on the cost-efficiency or resource planning. Fourth, the presence of a researcher (an observer) in the computer room while clinicians assess the patient scenarios might influence the results.

To sum up, the findings from this study protocol shall contribute to the development and implementation of a CDS system based on ML models for readmission reduction. The results of this study will be presented at scientific conferences, seminars with professional organizations, articles for media outlets, and submitted to a scientific peer-reviewed journal specialized in health technology.

## Appendix

Multimedia Appendix 1 Interview guide.

## Abbreviations

AI: artificial intelligence
CDS: clinical decision support
EHR: electronic health record
HF: heart failure
ICD-10: International Statistical Classification of Diseases, Tenth Revision
ICD-10-SE: Swedish version of the 10th revision of the International Classification of Diseases
ML: machine learning
SHAP: Shapley Additive Explanations

## References

[1] Qaddoura A, Yazdan-Ashoori P, Kabali C, Thabane L, Haynes RB, Connolly SJ, Van Spall HGC (2015). Efficacy of hospital at home in patients with heart failure: a systematic review and meta-analysis. PLoS One.

[2] Patil S, Johnson AE, Khera R, Chhatriwalla AK (2023). Socioeconomic disparities in readmission after heart failure hospitalization. Mayo Clin Proc.

[3] Nair M, Andersson J, Nygren JM, Lundgren LE (2023). Barriers and enablers for implementation of an artificial intelligence-based decision support tool to reduce the risk of readmission of patients with heart failure: stakeholder interviews. JMIR Form Res.

[4] Zhou H, Della PR, Roberts P, Goh L, Dhaliwal SS (2016). Utility of models to predict 28-day or 30-day unplanned hospital readmissions: an updated systematic review. BMJ Open.

[5] Ashfaq A, Sant'Anna A, Lingman M, Nowaczyk S (2019). Readmission prediction using deep learning on electronic health records. J Biomed Inform.

[6] Teo K, Yong CW, Chuah JH, Hum YC, Tee YK, Xia K, Lai KW (2023). Current trends in readmission prediction: an overview of approaches. Arab J Sci Eng.

[7] Soliman A, Agvall B, Etminani K, Hamed O, Lingman M (2023). The price of explainability in machine learning models for 100-day readmission prediction in heart failure: retrospective, comparative, machine learning study. J Med Internet Res.

[8] Hoffmann TC, Glasziou PP, Boutron I, Milne R, Perera R, Moher D, Altman DG, Barbour V, Macdonald H, Johnston M, Lamb SE, Dixon-Woods M, McCulloch P, Wyatt JC, Chan AW, Michie S (2014). Better reporting of interventions: Template for Intervention Description and Replication (TIDieR) checklist and guide. BMJ.

[9] Palinkas LA, Horwitz SM, Green CA, Wisdom JP, Duan N, Hoagwood K (2015). Purposeful sampling for qualitative data collection and analysis in mixed method implementation research. Adm Policy Ment Health.

[10] Ashfaq A, Lönn S, Nilsson H, Eriksson JA, Kwatra J, Yasin ZM, Slutzman JE, Wallenfeldt T, Obermeyer Z, Anderson PD, Lingman M (2020). Data resource profile: regional healthcare information platform in Halland, Sweden. Int J Epidemiol.

[11] Prokhorenkova L, Gusev G, Vorobev A, Dorogush AV, Gulin A (2018). CatBoost: unbiased boosting with categorical features. Adv Neur Inf Process.

[12] Lundberg SM, Erion G, Chen H, DeGrave A, Prutkin JM, Nair B, Katz R, Himmelfarb J, Bansal N, Lee SI (2020). From local explanations to global understanding with explainable AI for trees. Nat Mach Intell.

[13] Jacobs M, He J, Pradier F, Lam M, Ahn B, McCoy AC, Perlis RH, Doshi-Velez F, Gajos KZ (2021). Designing AI for Trust and Collaboration in Time-Constrained Medical Decisions: A Sociotechnical Lens. CHI '21: Proceedings of the 2021 CHI Conference on Human Factors in Computing Systems.

[14] McCoy A, Das R (2017). Reducing patient mortality, length of stay and readmissions through machine learning-based sepsis prediction in the emergency department, intensive care unit and hospital floor units. BMJ Open Qual.

[15] Sun TQ (2021). Adopting artificial intelligence in public healthcare: the effect of social power and learning algorithms. Int J Environ Res Public Health.

[16] Ji M, Genchev GZ, Huang H, Xu T, Lu H, Yu G (2021). Evaluation framework for successful artificial intelligence-enabled clinical decision support systems: mixed methods study. J Med Internet Res.

[17] Vanover C, Mihas P, Saldaña J (2021). Analyzing and Interpreting Qualitative Research: After the Interview, 1st Edition.

[18] Saldaña J (2015). The Coding Manual for Qualitative Researchers, 3rd Edition.

