# Supplementary material for: Machine Learning Model for Readmission Prediction of Patients With Heart Failure Based on Electronic Health Records: Protocol for a Quasi-Experimental Study for Impact Assessment
Source: JMIR Res Protoc. 2024 Mar 11;13:e52744. doi: 10.2196/52744 (PMC10964136; doi:10.2196/52744)
Supplement: Multimedia Appendix 1 [file resprot_v13i1e52744_app1.docx]

**Multimedia Appendix 1. Interview guide.**

Q1: Did the CDS model output provide any benefit for you or the patients?

Q2: How comfortable would you be to use the CDS model output in your everyday practice?

Q3: How confident were you to use the CDS model output? Did you think the risk score was reliable and valid?

Q4: What are possible unintended consequences of using CDS model output in the HF context?

Q5: How easy was it to understand the CDS model output?

Q6: What type of value could a CDS model output provide in your everyday practice?

Q7: Did you have all the information you needed to make a decision about the patients?

If No) what type of information was missing?

Q8: How close was this test to a real decision situation? Rate from 1 to 5.
